# Detection of recurrent high-grade glioma using microstructure characteristics of distinct metabolic compartments in a multimodal and integrative 18F-FET PET/fast-DKI approach

**DOI:** 10.1007/s00330-023-10141-0

**Published:** 2023-09-06

**Authors:** Johannes Lohmeier, Helena Radbruch, Winfried Brenner, Bernd Hamm, Brian Hansen, Anna Tietze, Marcus R. Makowski

**Affiliations:** 1 Department of Radiology, Charité - Universitätsmedizin Berlin, corporate member of Freie Universität Berlin and Humboldt Universität Zu Berlin, Charitéplatz 1, 10117 Berlin, Germany; 2 Department of Neuropathology, Charité - Universitätsmedizin Berlin, corporate member of Freie Universität Berlin and Humboldt Universität Zu Berlin, Charitéplatz 1, 10117 Berlin, Germany; 3 Department of Nuclear Medicine, Charité - Universitätsmedizin Berlin, corporate member of Freie Universität Berlin and Humboldt Universität Zu Berlin, Augustenburger Platz 1, 13353 Berlin, Germany; 4 Department of Clinical Medicine, Center of Functionally Integrative Neuroscience (CFIN), Aarhus University, Universitetsbyen 3, 8000 Aarhus C, Denmark; 5 Institute of Neuroradiology, Charité - Universitätsmedizin Berlin, corporate member of Freie Universität Berlin and Humboldt Universität Zu Berlin, Charitéplatz 1, 10117 Berlin, Germany; 6 Department of Radiology, Technical University Munich, Ismaninger Str. 22, 81675 München, Germany

**Keywords:** Glioma, Metabolism, Positron-emission tomography, Multimodal imaging, Diffusion magnetic resonance imaging

## Abstract

**Objectives:**

Differentiation between high-grade glioma (HGG) and post-treatment-related effects (PTRE) is challenging, but advanced imaging techniques were shown to provide benefit. We aim to investigate microstructure characteristics of metabolic compartments identified from amino acid PET and to evaluate the diagnostic potential of this multimodal and integrative O-(2-^18^F-fluoroethyl)-l-tyrosine-(FET)-PET and fast diffusion kurtosis imaging (DKI) approach for the detection of recurrence and IDH genotyping.

**Methods:**

Fifty-nine participants with neuropathologically confirmed recurrent HGG (*n* = 39) or PTRE (*n* = 20) were investigated using static ^18^F-FET PET and a fast-DKI variant. PET and advanced diffusion metrics of metabolically defined (80–100% and 60–75% areas of ^18^F-FET uptake) compartments were assessed. Comparative analysis was performed using Mann–Whitney *U* tests with Holm-Šídák multiple-comparison test and Wilcoxon signed-rank test. Receiver operating characteristic (ROC) curves, regression, and Spearman’s correlation analysis were used for statistical evaluations.

**Results:**

Compared to PTRE, recurrent HGG presented increased ^18^F-FET uptake and diffusivity (MD60), but lower (relative) mean kurtosis tensor (rMKT60) and fractional anisotropy (FA60) (respectively *p* < .05). Diffusion metrics determined from the metabolic periphery showed improved diagnostic performance — most pronounced for FA60 (AUC = 0.86, *p* < .001), which presented similar benefit to ^18^F-FET PET (AUC = 0.86, *p* < .001) and was negatively correlated with amino acid uptake (*r*s =  − 0.46, *p* < .001). When PET and DKI metrics were evaluated in a multimodal biparametric approach, TBRmax + FA60 showed highest diagnostic accuracy (AUC = 0.93, *p* < .001), which improved the detection of relapse compared to PET alone (difference in AUC = 0.069, *p* = .04). FA60 and MD60 distinguished the IDH genotype in the post-treatment setting.

**Conclusion:**

Detection of glioma recurrence benefits from a multimodal and integrative PET/DKI approach, which presented significant diagnostic advantage to the assessment based on PET alone.

**Clinical relevance statement:**

A multimodal and integrative ^18^F-FET PET/fast-DKI approach for the non-invasive microstructural characterization of metabolic compartments provided improved diagnostic capability for differentiation between recurrent glioma and post-treatment-related changes, suggesting a role for the diagnostic workup of patients in post-treatment settings.

**Key Points:**

*• Multimodal PET/MRI with integrative analysis of* ^*18*^ *F-FET PET and fast-DKI presents clinical benefit for the assessment of CNS cancer, particularly for the detection of recurrent high-grade glioma.*

*• Microstructure markers of the metabolic periphery yielded biologically pertinent estimates characterising the tumour microenvironment, and, thereby, presented improved diagnostic accuracy with similar accuracy to amino acid PET.*

*• Combined* ^*18*^ *F-FET PET/fast-DKI achieved the best diagnostic performance for detection of high-grade glioma relapse with significant benefit to the assessment based on PET alone.*

**Supplementary information:**

The online version contains supplementary material available at 10.1007/s00330-023-10141-0.

## Introduction

Cerebral gliomas are the most common primary brain tumours in adults. Despite comprehensive therapeutic regimens and significant advances in treatment strategies in recent years [1], the survival prospects for patients with high-grade glioma (HGG) remain poor due to a high risk for recurring disease. The glioma microenvironment, characterized by its complex molecular pathophysiology, dynamic plasticity and multifaceted cellular interactions [2–5], plays a central role in several aspects of cancer progression, including diffuse infiltration, drug resistance and immune escape [6].

Early recognition of relapsing glioma is difficult, but crucial for patient survival, as prompt medical intervention may change the otherwise dismal prognosis. Given the potential for perioperative complications, neurosurgical biopsies are considered a last resort for the diagnosis of recurrence. Moreover, the accuracy of diagnosis is highly dependent on accurate targeting in often heterogeneous lesions, where benign treatment-associated changes may be co-existent. Contrast-enhanced magnetic resonance imaging (CE-MRI) is the clinical standard for the initial diagnosis and surveillance of brain tumours, but clinical imaging provides poor sensitivity for the detection of recurrence, as post-treatment-related effects (PTRE) — e.g. radiation effects, necrosis, neuroinflammation or postsurgical changes — are often indistinguishable [7]. Definition of tumour boundaries based on CE-MRI is furthermore known to underestimate tumour margins, due to a well-known mismatch between contrast-enhancing regions and diffuse infiltration [8]. Particularly in the post-treatment setting, where concurrent therapy-associated changes may be apparent, CE-MRI becomes less reliable [7]. Unlike gadolinium-based contrast media, metabolic imaging using O-(2-^18^F-fluoroethyl)-l-tyrosine (FET), an established amino acid tracer with a clinical role in the diagnosis, tumour grading, treatment planning, and follow-up imaging of primary brain tumours [9–12], is independent of blood–brain barrier (BBB) impairment, enabling a more accurate determination of the tumour extent [7, 13–15].

Besides conventional CE-MRI, diffusion MRI (dMRI) emerged as an important clinical application in cancer imaging, which determines the diffusion properties of protons within tissue, and, thereby, provides an indirect probe of the cellular-level tissue architecture. By taking a more complex diffusion profile into account — beyond the scope of traditional diffusion-weighted (DWI) and diffusion tensor (DTI) imaging — a greater microstructural sensitivity towards alterations of tissue composition is achieved using diffusion kurtosis imaging (DKI) [16]. However, further clinical evaluation and translation of DKI have been impeded by time-consuming acquisitions and computationally demanding kurtosis tensor analysis. To alleviate these demands, a fast-DKI method was recently proposed [17]. In this novel DKI variant, estimates from (non-)Gaussian diffusion are rapidly obtained from 19 diffusion-weighted images at a fraction of the measurement time, which renders this technique suitable for routine diagnostic imaging, particularly when prolonged measurements cannot be endured (with motion artefacts or early termination of the examination as consequence) or in clinical hybrid PET/MRI examinations where tracer decay excludes extensive acquisition protocols.

**Fig. 1:**
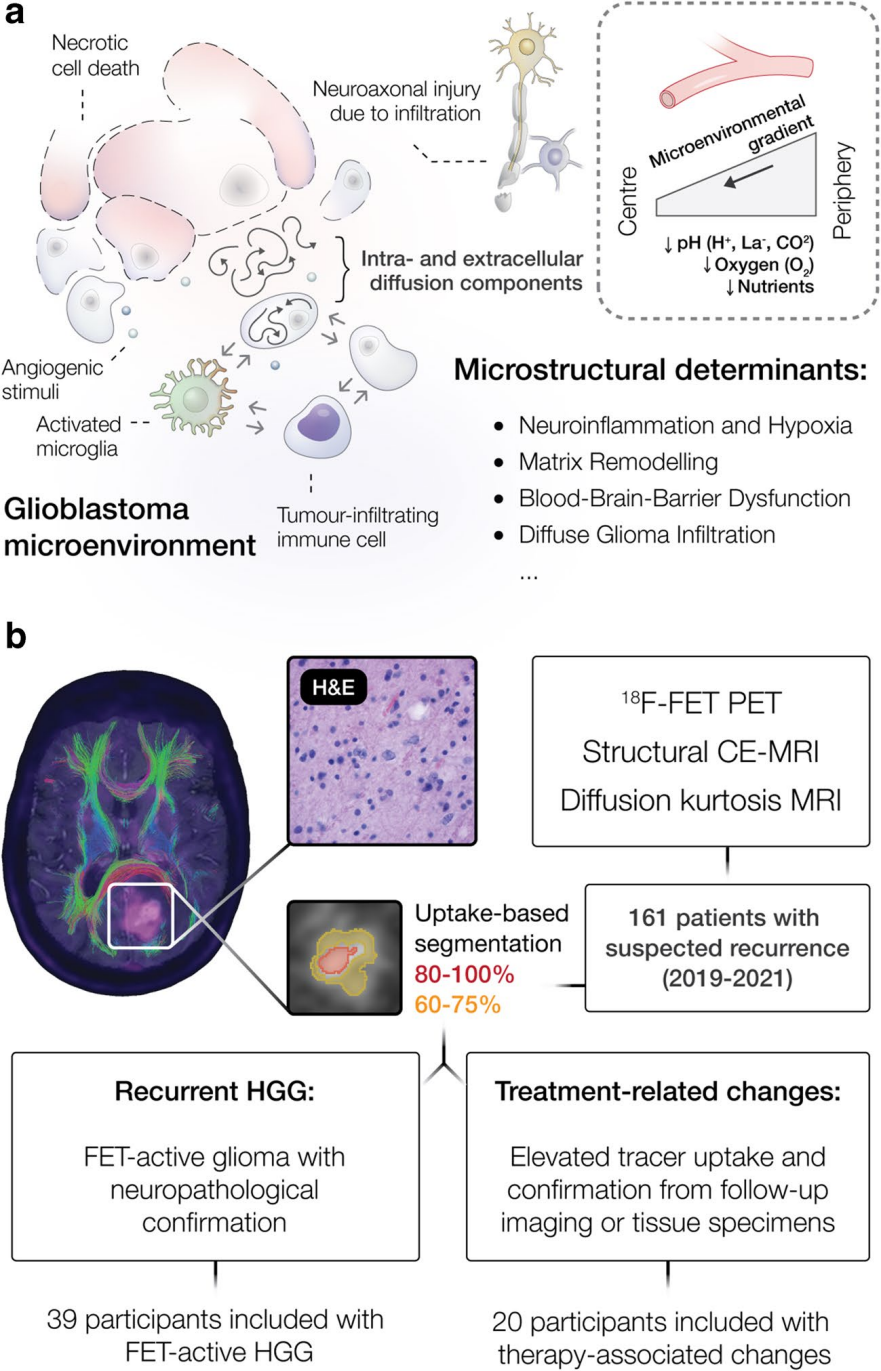
Microstructural disarray in the glioblastoma microenvironment. Advanced diffusion techniques can determine microstructural tissue properties (**a**) in relapsing cancer. Due to a dysregulated cancer metabolism, the glioblastoma microenvironment is transformed into a hypoxic, acidic and nutrient-depleted milieu. While central regions of highly aggressive tumours typically present necrotic features, the periphery shows higher proliferation rates due to increased vascularity in response to angiogenic stimuli and, therefore, improved nutrient/oxygen supply and metabolic waste disposal. Blood–brain barrier (BBB) impairment is accompanied by vasogenic oedema, deposition of plasma proteins or the occurrence of haemorrhage at microvascular disruption. Tumour infiltrating leucocytes aggregate in the tumour parenchyma and periphery, where a distinct immune microenvironment of CNS-resident and peripheral immune cells is established, that can inhibit or promote cancer development and growth. Multimodal data from hybrid PET/MRI (see **b**) combining CE-MRI, ^18^F-FET PET and DKI — acquired in a single session in clinically practicable acquisition time — is demonstrated in recurrent glioma, which presents a mismatch between contrast enhancement and ^18^F-FET-active tumour. As amino acid PET is known to provide more accurate information on the tumour extent compared to CE-MRI, we determined microstructural diffusion markers in metabolic compartments defined by ^18^F-FET uptake in a combined and integrative PET/DKI approach. Haematoxylin and eosin (H&E) staining from the same patient confirms recurrence (magnification 400-fold). Patients with suspected cancer relapse that received concurrent PET/MRI between 2019 and 2021 were retrospectively investigated. BBB, blood–brain barrier; HGG, high-grade glioma

Although several advanced MRI techniques have been investigated to characterize structural, metabolic, or vascular features of brain tumours, the diagnostic performance of PET and MRI alone remain restricted. With the increasing clinical availability of hybrid PET/MRI systems and recommendations for the synergistic use of amino acid PET and MRI [18], there is urgent need for the development of neuroimaging techniques that integrate metabolic and microstructural data into biological determinants, characterising the heterogenous tumour environment (see Fig. 1, panel a), and, thereby, guide precision diagnostics and the non-invasive detection of cancer. Therefore, we established a methodology for combined and integrative multimodal PET/MRI analysis, targeting the microstructural characterization of the metabolically active tumour environment. Using metabolic imaging for the definition of ^18^F-FET-active tumour margins (see Fig. 1, panel b), we investigated microstructural diffusion markers of distinct metabolic compartments and evaluated the diagnostic potential of this integrative PET/DKI approach for the detection of glioma relapse and IDH genotyping in a patient cohort previously treated for glioma.

## Patients and methods

### Study design and patients

In this retrospective clinical cohort study, consecutive patients with previously treated glioma, referred for PET/MRI due to suspected recurrence between 2019 and 2021, were investigated using concurrent multimodal O-(2-^18^F-fluoroethyl)-l-tyrosine (FET) PET, CE-MRI and fast-DKI. Participants with newly diagnosed ^18^F-FET-active lesions were included according to the eligibility criteria, as shown in Fig. 1, panel b. Neuropathological confirmation or follow-up in line with the Response Assessment in Neuro-Oncology (RANO) criteria [19] was used as reference. This study was conducted in accordance with the ethical standards of the institutional and national research committee and with the 1964 Helsinki Declaration and its later amendments. Approval from the institutional ethics board was obtained (EA2/019/23). Informed consent was obtained from all patients included in the study.

### Neuropathological analysis

Glioma recurrence was classified according to the latest revision of the World Health Organization (WHO) classification of tumours of the CNS [20] (according to the molecular data available at that time point). Molecular characteristics of IDH mutation (IDH-MT/WT, IDH-mutant/wildtype), 1p/19q co-deletion (LOH1p/19q + , co-deleted, LOH1p/19q-, non-deleted), O6-methylguanine-DNA-methyltransferase promoter methylation (MGMT + , methylated, MGMT-, unmethylated) and alpha-thalassemia/mental retardation syndrome X-linked loss (ATRX + , deficient, ATRX-, retention) were determined from formalin-fixed paraffin-embedded tissue specimens during routine diagnostic procedures based on fluorescence in situ hybridisation, pyrosequencing, EPIC DNA methylation arrays or immunostaining. MGMT promoter methylation status was determined by pyrosequencing with an established cutoff value of ≥ 10%, differentiating methylated vs. unmethylated cases [21] or EPIC DNA methylation array. Molecular status was determined ex domo in one instance. An overview of the molecular stratification is available in Table 1 and in Appendix E1 (online), Table 1.

**Table 1:** Summarised characteristics of the study cohort. *NA*, not applicable/available; *WHO*, World Health Organization; *IDH*, isocitrate dehydrogenase; *MGMT*, O6-methylguanine-DNA-methyltransferase; *ATRX*, alpha-thalassemia/mental retardation syndrome X-linked; *LOH1p/19q*, loss of heterozygosity (LOH) of 1p/19q

	Entire cohort	Recurrent glioma	Post-treatment-related changes
Participants (*n*)	59	39	20
Age (M ± SD in a)	48 ± 12	48 ± 12	48 ± 11
Gender (male/female)	29/30	19/20	10/10
WHO tumour classification (II°/III°/IV°)	7/24/28	0/16/23	7/8/5
LOH1p/19q (negative/positive/NA)	24/16/19	20/7/12	4/9/7
IDH mutation status (negative/positive/NA)	24/35/0	19/20/0	5/15/0
MGMT promoter methylation status (negative/positive/NA)	15/34/10	13/19/7	2/15/3
ATRX loss (negative/positive/NA)	38/17/4	25/11/3	13/6/1

### Imaging and pre-processing

Simultaneous PET/MR imaging was performed on a MAGNETOM Biograph mMR (Siemens Healthcare) [22]. Static PET and clinical high-field (3 T) MRI were acquired in list mode for up to 60 min after intravenous (i.v.) administration of ^18^F-FET (M ± SD, 163 ± 23 MBq; standard-dose 180 MBq and individually calculated dose for body weight < 60 kg). Gadolinium-based contrast agent (Gadovist®, Bayer Pharma AG) was administered according to the patient’s total body weight (0.1 mmol/kg). Fasting for at least 4 h before PET acquisition was recommended [23].

The MRI acquisition protocol included a transversal T1-weighted ultrashort echo time (UTE) for attenuation and scatter correction, a T2-weighted (TR/TE = 5320/88 ms; matrix size = 230 × 230 × 230; voxel size = 0.4 × 0.4 × 3.0 mm^3^), a post-contrast T1-weighted magnetization-prepared rapid gradient echo (TR/TE/TI = 2400/2.26/900 ms; flip angle = 8°; matrix size = 256 × 256 × 256; voxel size = 1.0 × 1.0 × 1.0 mm^3^; thickness = 1 mm; slices = 192) and a rapid (approx. 3 min) DKI (TR/TE = 9200/111 ms; matrix size = 342 × 342 × 342; voxel size = 3.6 × 3.6 × 2.5 mm^3^; thickness = 2.5 mm; slices = 50; diffusion scheme = bipolar; *b*-values = 0 s/mm^2^, 1000 s/mm^2^, 2500 s/mm^2^; echo spacing = 0.68 ms; EPI factor = 96; bandwidth = 1578 Hz/Px) sequence with distinct diffusion encoding directions and a custom gradient table [17].

PET acquisition was reconstructed into transaxial slices using an iterative ordered subset expectation maximisation algorithm (OSEM, 3 iterations and 21 subsets; matrix size = 344 × 344 × 127; voxel size = 1.0 × 1.0 × 2.3 mm^3^; gaussian filter = 3 mm). Emission data was corrected for decay, randoms, dead time, scatter and attenuation. Summed images over a time frame of 20 min p.i. were used for evaluation of PET.

Pre-processing of medical images included a denoising algorithm [24], Gibbs artefacts correction [25], eddy-currents correction and adjustment for patient motion using FMRIB’s Software Library (FSL, v6.0.3) [26] and MRtrix (v3) [24, 26, 27] as well as bias-field correction using the N4 method [28]. Following these steps, medical images were visually inspected to ensure sufficient data quality prior to DKI calculation. Using the calculation methods for the 1–9-9 scheme introduced by Hansen et al [17], a robust mean kurtosis estimate (MKT), mean diffusivity (MD) and fractional anisotropy (FA) were computed using a generally available software in Matlab 2020a (Mathworks).

### PET and MRI analysis

Quantitative analysis was performed using OsiriX MD 12 (Pixmeo SARL) using matched multimodal datasets. Where applicable, the most prominent lesion with greatest lesion size, ^18^F-FET tracer uptake and contrast enhancement, closest to the resection cavity or primary tumour location was chosen as target lesion. Segmentation was performed using automated 3D iso-contouring based on ^18^F-FET tracer uptake (J.L. and M.M., 6 and > 10 years of experience in MR imaging analysis), yielding a metabolically defined centre (80–100%, ROI80) and peripheral (60–75%, ROI60) 3D region-of-interest. In order to account for non-specific and regional uptake behaviour, mean and maximum target-to-background (TBRmean, TBRmax) ratios were determined from the standardized uptake value (SUV) measured in ROI80 and the mean background uptake in the unaffected contralateral hemisphere, which was determined using a 2D ROI with similar size (in the slice with highest mean uptake within the 3D ROI).

Where applicable, PET-derived ROIs were marginally adapted to avoid fluid compartments (e.g. resection cavities and macrocystic components) or large blood vessels in order to reflect solid tumour components. 3D ROIs were then transformed to DKI space using non-rigid deformation (ANTs, v2.3.4) [29]. Due to the distinct regional differences in non-Gaussian diffusion, MKT was normalised as a ratio between the mean diffusion signal from the lesion and the corresponding diffusion signal in the unaffected contralateral hemisphere. Quantitative analysis was performed for each scalar map (MD, MKT, FA) using both ROIs (ROI60, ROI80), yielding metrics for each metabolic compartment (MD60/MD80; rMKT60/rMKT80; FA60/FA80).

### Statistics

Statistical analysis was performed using Prism v.9 (GraphPad Software) and MedCalc v20.104 (MedCalc Software Ltd). The Mann–Whitney *U* (two-tailed) tests (with Holm-Šídák’s multiple-comparison test) were used for comparisons between two groups. The Wilcoxon signed-rank (two-tailed) test was used for matched pairs. Receiver operating characteristic (ROC) analysis was performed using the DeLong method reporting area under the curve (AUC), 95% confidence intervals (CI) and *p*-value. Sensitivity and specificity were reported for the best cutoff point independent from prevalence determined using Youden’s index [30]. Logistic regression was used to model binary outcome. Measurements were correlated and evaluated using the Spearman correlation coefficient. *p*-value < 0.05 was considered statistically significant. M = Mean. SD = Standard deviation. SE = Standard error. Mdn = Median. IQR = Interquartile range.

## Results

### Patient cohort

In summary, 59 patients (29 men, 30 women; age, M ± SD, 48 a ± 12) were retrospectively identified. Recurrent HGGs (*n* = 39) were histopathologically validated upon neurosurgical intervention comprising 16 WHO grade 3 and 23 WHO grade 4 tumours. PTRE (*n* = 20) was confirmed by follow-up imaging with a median post-treatment surveillance of 21.50 months [IQR, 9.75 mo] or neuropathological reports (10%). Median time interval between last intervention scheme and PET/MRI examination was 30 months [IQR, 49 mo]. An overview of the molecular stratification is available in Table 1. Additional information is available in Appendix E1 (online), Table 1.

### Evaluation of static ^18^F-FET PET and DKI

**Fig. 2:**
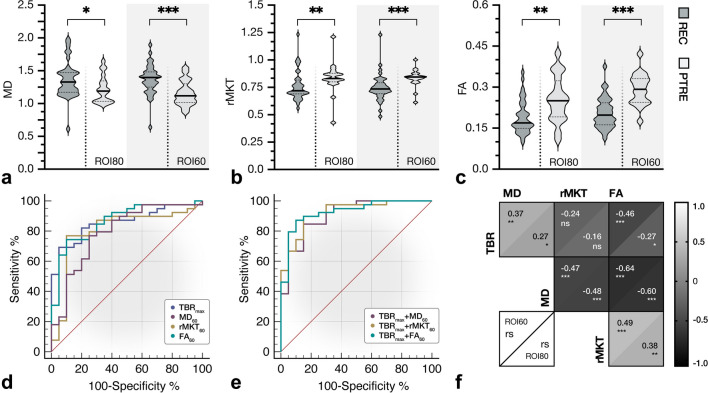
Statistical analysis of ^18^F-FET PET and DKI. Comparative statistics of diffusion metrics are shown in panels **a**–**c**. Compared to treatment-related changes, recurrence presented higher MD, lower rMKT and a decrease in FA. Receiver operating characteristic (ROC) analysis (**d** and **e**) demonstrated that TBRmax (AUC ± SE, 0.86 ± 0.05, *p* < .001) and FA60 (AUC ± SE, 0.86 ± 0.05, *p* < .001) showed the best performance in univariate analysis, while biparametric assessment of TBRmax + FA60 presented highest diagnostic performance (AUC ± SE, 0.93 ± 0.04, *p* < .001) with additional diagnostic benefit (difference in AUC ± SE, 0.069 ± 0.034, *p* = .04). **f **Correlative evaluation of ^18^F-FET PET and DKI showed notable correlation of amino acid uptake with FA60 (*r*s =  − 0.46, *p* < .001) and MD60 (*r*s = 0.37, *p* = .004) within the peripheral metabolic compartment. Peripheral metabolic compartments presented slightly increased Spearman’s correlation coefficients. *p*-value < .05 was considered statistically significant. (*) *p*-value < .05, (**) *p*-value < .01, (***) *p*-value < .001, (ns) non-significant

Recurrent HGG demonstrated higher mean (TBRmean, Mdn, 2.41 [IQR, 1.15, *n* = 39] vs. 1.46 [IQR, 0.32, *n* = 20], *p* < 0.001, *U* = 109) and maximum (TBRmax, Mdn, 2.86 [IQR = 1.60, *n* = 39] vs. 1.72 [IQR, 0.46, *n* = 20], *p* < 0.001, *U* = 111) ^18^F-FET tracer uptake compared to treatment effects, which presented reliable diagnostic performance (AUC ± SE, 0.86 ± 0.05, *p* < 0.001) for the differentiation between relapse and therapy-associated changes, as shown in Table 2 and Fig. 2, panel d. A difference in lesion volume between metabolic compartments was apparent (Mdn, 0.59 cm^3^ [IQR, 0.68] (ROI80) vs. 2.38 cm^3^ [IQR, 3.72] (ROI60), *p* < 0.001, *n* = 59, W = 1770), while there was no substantial difference (*p* > 0.05) between recurrent HGG and PTRE.

**Table 2:** Diagnostic measures from ^18^F-FET PET and DKI. Among diffusion metrics, FA60 showed highest diagnostic power with similar diagnostic benefit to ^18^F-FET PET. No difference in (SUV and MKT) background signal was apparent between both groups (*p* > .05). *p*-value < .05 was considered statistically significant. *ROC*, receiver operating characteristic; *AUC*, area under the curve; *DTI*, diffusion tensor imaging; *DKI*, diffusion kurtosis imaging

		Mann–Whitney *U* test	ROC analysis	
	Metric	Adjusted *p*-value, mean rank difference, MWU	*p*-value, AUC ± SE, 95% CI	Threshold, sensitivity/specificity
^18^F-FET PET Target-to-background ratio	TBRmean_80_ (TBRmean)	*p* < .001, 21.26, *U* = 109	*p* < .001, AUC = 0.86 ± 0.05, 95% CI = 0.75–0.94	> 1.73, 82%/85%
	TBRmax_80_ (TBRmax)	*p* < .001, 21.10, *U* = 111	*p* < .001, AUC = 0.86 ± 0.05, 95% CI = 0.74–0.94	> 2.42, 69%/95%
Diffusivity	MDmean_80_ (MD80)	*p* = .034, 11.19, *U* = 242	*p* = .014, AUC = 0.69 ± 0.08, 95% CI = 0.56–0.80	> 1.05, 97%/40%
	MDmean_60_ (MD60)	*p* < .001, 17.25, *U* = 162	*p* < .001, AUC = 0.79 ± 0.07, 95% CI = 0.67–0.89	> 1.24, 77%/75%
Kurtosis	rMKTmean_80_ (rMKT80)	*p* = .008, − 13.92, *U* = 206	*p* < .001, AUC = 0.74 ± 0.07, 95% CI = 0.60–0.84	≤ 0.78, 72%/90%
	rMKTmean_60_ (rMKT60)	*p* < .001, − 17.93, *U* = 153	*p* < .001, AUC = 0.80 ± 0.07, 95% CI = 0.68–0.90	≤ 0.79, 77%/90%
Fract. anisotropy	FAmean80 (FA80)	*p* = .003, − 14.60, *U* = 197	*p* < .001, AUC = 0.75 ± 0.07, 95% CI = 0.62–0.85	≤ 0.21 74%/70%
	FAmean60 (FA60)	*p* < .001, − 21.48, *U* = 106	*p* < .001, AUC = 0.86 ± 0.05, 95% CI = 0.75–0.94	≤ 0.23, 74%/90%
Biparametric ^18^F-FET PET/DKI	TBRmax_80_ + MDmean_60_	-	*p* < .001, AUC = 0.91 ± 0.04, 95% CI = 0.80–0.97	85%/85%
	TBRmax_80_ + rMKTmean_60_	-	*p* < .001, AUC = 0.92 ± 0.04, 95% CI = 0.82–0.98	90%/85%
	TBRmax_80_ + FAmean_60_	-	*p* < .001, AUC = 0.93 ± 0.04, 95% CI = 0.83–0.98	87%/90%

**Fig. 3:**
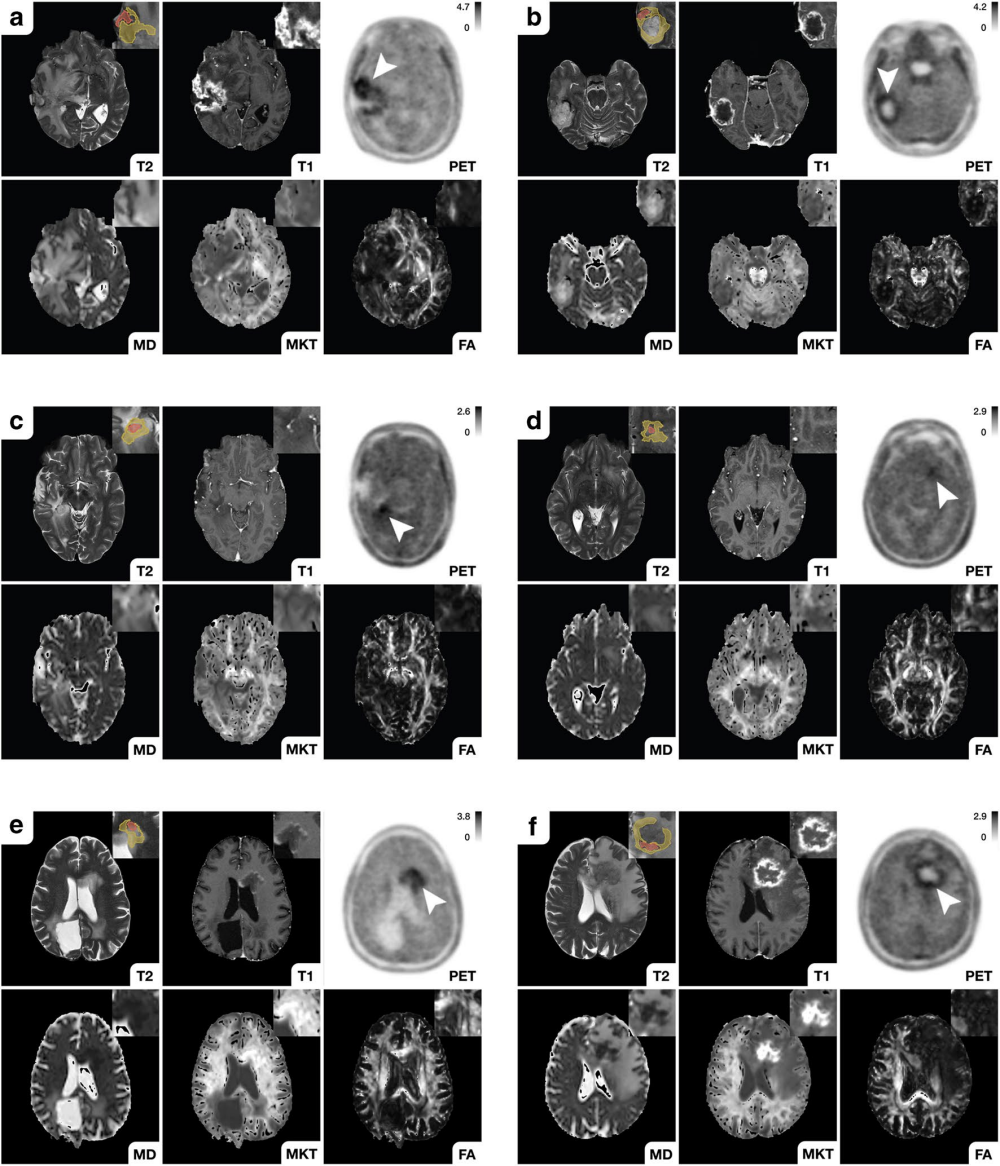
Diffusion characteristics of recurrent glioma. Recurrent HGG typically presented an increase in MD as well as a decrease in rMKT and FA in both metabolic tumour compartments (see **a**–**d**). ^18^F-FET-active lesions are indicated by the white arrow (first row, last column). Magnified insets are shown at the top right. Both metabolic compartments (ROI60, yellow; ROI80, red) are illustrated (the first row, first column). Panel **a** shows an extensive glioblastoma recurrence (CNS WHO grade 4, IDH-WT, MGMT + , ATRX-) with high ^18^F-FET uptake (TBRmax, 3.5) and considerable gadolinium-based contrast enhancement. Histopathology revealed an astroglial tumour with mixed radiogenic, reactive and regressive changes. **b** Recurrent glioblastoma (CNS WHO grade 4, MGMT + , IDH-WT, ATRX-) surrounding the resection cavity. A surrounding rim (see peripheral metabolic compartment) with elevated tracer uptake (TBRmax, 3) and contrast enhancement, which was later shown to correspond to vital tumour cells mixed with radiogenic features and signs of diffuse haemorrhage. Diffusion metrics in this area presented high MD, decreased rMKT and low FA. **c** Localized ^18^F-FET uptake (TBRmax, 2.5) corresponding to elevated MD and decreased rMKT that was confirmed to be a recurrent astrocytoma (CNS WHO grade 3, IDH-MT, MGMT-, ATRX + , LOH1p/19q-). In panel **d**, a small non-enhancing lesion with increased ^18^F-FET uptake (TBRmax, 2.1) is demonstrated in a patient with multifocal recurrence. Lesion size and ^18^F-FET uptake might be underestimated in PET due to partial volume effects. Diffusion metrics showed increased MD, low rMKT and FA, suggesting recurrence. Panels **e** and **f** exemplify less common clinical presentations of recurrent glioma with low MD and increased rMKT. A recurrent astrocytoma (CNS WHO grade 4, IDH-MT, MGMT + , ATRX + , LOH1p/19q-) distant to the primary resection site (see **e**) is illustrated with high ^18^F-FET uptake (TBRmax, 4), low MD and elevated rMKT. Histopathology showed a glial tumour with high proliferation rate in the absence of necrotic features. High cellular (mutant) P-53 accumulation was reported, which is known to have an impact on the expression of various cancer-related genes, suggesting hindered diffusion also as a result of dysregulated protein synthesis. **f** Recurrent oligodendroglioma (CNS WHO grade 3, LOH1p/19q + , IDH-MT, ATRX-) close to the resection site (not shown in the axial slice) with extensive necrosis and a contrast-enhancing edge with elevated ^18^F-FET tracer uptake (TBRmax, 2.8). The first biopsy revealed radionecrosis, but a second biopsy 6 months later confirmed recurrence with vital tumour cells, reactive/regressive CNS tissue and necrosis. Although a large fraction of the spherical mass presented elevated rMKT and low MD, diffusion metrics sampled from the metabolically active regions showed inverse signal behaviour with high MD and low rMKT, already suggesting cancer relapse at imaging prior to the first biopsy

**Fig. 4:**
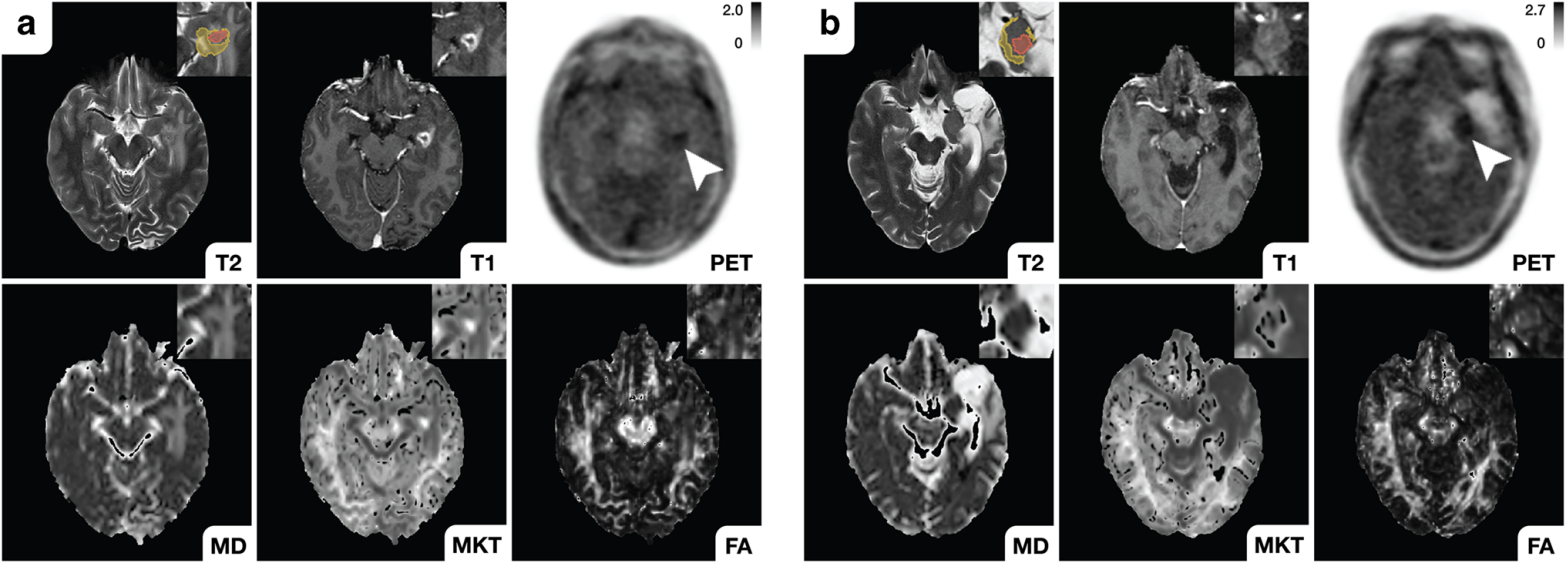
Imaging of treatment-related changes. Clinical presentations of histopathologically confirmed PTRE are illustrated. ^18^F-FET-active lesions are indicated by the white arrow (first row, last column). Magnified insets are shown at the top right. Both metabolic compartments (ROI60, yellow; ROI80, red) are illustrated (the first row, first column). First panel (**a**) shows a patient with a previously treated anaplastic pleomorphic xanthoastrocytoma (CNS WHO grade 3, LOH1p/19q-, IDH-WT, MGMT-, ATRX-), who presented with a new focal contrast-enhancing lesion with low ^18^F-FET uptake (TBRmax, 1.7). Following primary resection, the patient received a combined therapy according to the Stupp’s regimen. Upon first relapse, a second-line therapy was started with BRAF/MEK inhibitors and radiotherapy. The new lesion was histopathologically confirmed to be radionecrosis with moderate reactive tissue changes. Diffusion metrics showed increased rMKT and low MD, suggesting microstructural complexity. A patient with a progressive hippocampal lesion (see **b**) with low ^18^F-FET tracer uptake (TBRmax, 1.6) is demonstrated, who was previously treated for an oligodendroglioma (CNS WHO grade 3, LOH1p/19q + , IDH-MT, ATRX-) with resection and concomitant radiochemotherapy (CCNU/PCV). Histopathology showed reactive CNS changes without malignancy or active inflammation. Diffusion characteristics were similar to the unaffected contralateral hemisphere indicating low microstructural disarray

Quantitative diffusion metrics sampled from the metabolic periphery (see ROI60) presented greater distinction between diagnostic groups (see Table 2 and Fig. 2). Most notably, in the peripheral metabolic region, relapsing glioma presented higher MD (Mdn, 1.40 [IQR, 0.23, *n* = 39] vs. 1.12 [IQR, 0.29, *n* = 20], *p* < 0.001, *U* = 162), lower rMKT (Mdn, 0.736 [IQR, 0.100, *n* = 39] vs. 0.844 [IQR, 0.060, *n* = 20], *p* < 0.001, *U* = 153) and decreased FA (Mdn, 0.198 [IQR, 0.081, *n* = 39] vs. 0.292 [IQR, 0.087, *n* = 20], *p* < 0.001, *U* = 106) than PTRE, as exemplified in Figs. 3 and 4. On the opposite, neuropathologically described (radio-)necrosis (see Fig. 3, panel f and Fig. 4, panel a) demonstrated an inverse signal behaviour with an increase in rMKT and decreased MD. Interestingly, the study comprised a case of a highly proliferative glioblastoma distant to the primary resection site, which presented diffusion restriction and an increase in rMKT, as shown in Fig. 3, panel e. No differences between PTRE with previously treated HGG and LGG (respectively *p* > 0.05) were observed.

Metabolic compartments showed disparate diagnostic performances, which was most pronounced for FA and MD (see Table 2). Among diffusion metrics, FA60 presented highest diagnostic power (AUC ± SE, 0.86 ± 0.05, *p* < 0.001), essentially yielding similar benefit to PET, while rMKT60 (AUC ± SE, 0.80 ± 0.07, *p* < 0.001) and MD60 (AUC ± SE, 0.79 ± 0.07, *p* < 0.001) presented moderate discriminative capability, as illustrated in Fig. 2, panel d. When DKI-derived metrics and ^18^F-FET PET were evaluated in a multimodal biparametric approach (see Fig. 2, panel e), highest AUC was apparent for the clinically established TBRmax combined with FA60 (AUC ± SE, 0.93 ± 0.04, *p* < 0.001), which improved the diagnostic performance compared to PET alone (difference in AUC ± SE, 0.069 ± 0.034, *p* = 0.04).

### Correlation of metabolic and diffusion metrics

The Spearman correlation coefficients showed a trend towards slightly increased correlation for metrics from the metabolic periphery. Notably, ^18^F-FET uptake in the peripheral metabolic compartment was correlated with FA60 (*r*s =  − 0.46, *p* < 0.001) and MD60 (*r*s = 0.37, *p* = 0.004), as shown in Fig. 2, panel f. As expected, a moderate to strong association was apparent between (non-)Gaussian diffusion metrics.

### IDH genotype and diffusion properties

IDH mutation was discriminated from wildtype both in the entire cohort (AUC ± SE, 0.78 ± 0.06, *p* < 0.001) and in a subgroup analysis of recurrent HGG (AUC ± SE, 0.74 ± 0.08, *p* = 0.005) at MD60 ≤ 1.29. In participants with PTRE, IDH mutation was distinguished likewise at lower MD60 (AUC ± SE, 0.83 ± 0.10, *p* = 0.002) and at higher FA60 (AUC ± SE, 0.84 ± 0.12, *p* = 0.003), suggesting less severe tissue damage in the post-treatment situation.

## Discussion

We demonstrated that the detection of recurrent glioma benefits from a multimodal and integrative PET/DKI approach using diffusion microstructure markers of distinct compartments identified from metabolic imaging. We showed that glioma relapse elicits characteristic changes in diffusion properties that can be used to distinguish recurrence from treatment effects in previously treated glioma. Diffusion metrics determined from the metabolic periphery yielded biologically pertinent estimates characterising the tumour microenvironment, and, thereby, presented improved diagnostic performance for the differentiation between recurrent glioma and therapy-associated changes — most notably for FA, which showed similar diagnostic benefit to ^18^F-FET PET. When ^18^F-FET PET and DKI metrics were evaluated in a multimodal biparametric approach, this could be used to achieve improved diagnostic accuracy and an additional diagnostic benefit to the assessment based on PET alone.

(Non-)Gaussian diffusion metrics from DKI are highly sensitive to the tissue microstructure [31]. Low FA was apparent in recurrent HGG, suggesting compromised fibre integrity, such as in diffuse infiltration. When FA — as an index of the directional variation of diffusivity — is decreased, diffusion is almost equal in all directions, whereas an increase indicates that distinct directions show faster diffusion (e.g. along intact fibre tracts). Due to a highly dysregulated amino acid metabolism in HGG [32] and a destructive infiltrative growth pattern with extensive neuroaxonal injury [33], FA60 demonstrated reliable diagnostic performance and a moderate negative correlation with ^18^F-FET tracer uptake. Consistently, a previous study reported that decreased peritumoural FA in pre-surgical glioblastoma predicted later recurrence due to an underestimation of tumour extent based on structural CE-MRI [34]. Our findings suggest that the assessment of perifocal FA for the differentiation between relapse and treatment-related changes might be a viable option for follow-up imaging, particularly in circumstances where PET is unavailable, considering that DTI protocols are widely accessible and clinically used for DTI fibre tracking-based neuronavigation. Pre-operative and radiation planning may further benefit from the integration of diffusion microstructure markers, as there is potential to detect diffuse infiltration beyond the margins of contrast enhancement.

Recurrent HGG showed decreased rMKT, consistent with less diffusion compartmentalization as in microstructural damage and cellular loss. Our findings indicate a role for the identification of (radio-)necrosis, as we observed elevated rMKT in neuropathologically confirmed necrosis corresponding to microstructural complexity. Moreover, there was a case of a recurrent glioblastoma with high proliferation rate distant to the primary resection site — presumably less affected from previous treatment efforts — which demonstrated an atypical signal behaviour with increased rMKT, suggesting high cellularity with a more restrictive and compartmentalized diffusion environment.

We found increased MD in recurrence, which could indicate the presence of vasogenic oedema, altered vascularity or inflammatory changes. A restricted diffusion component, assumed to be caused by high cellularity in HGG, might be obfuscated by these concurrent pathophysiological effects in previously treated glioma. Considering that DKI is known to provide more accurate estimates of Gaussian diffusion metrics [35], the observed high variability of MD suggests that a directionally averaged diffusivity might be less robust for the diagnostic differentiation between recurrent HGG and PTRE.

With the recent revision of the World Health Organization (WHO) classification of tumours for the central nervous system [36], there has been considerable interest in molecular markers, which now play a central role for the integrated classification of CNS cancers. IDH mutation status, as an important clinical marker for molecular targeting and prognosis [37], was reflected in microstructural diffusion properties. Higher FA60 and lower MD60 distinguished IDH mutation in patients with previously treated gliomas, suggesting less radical microstructural tissue damage in the post-treatment situation, which could be a result of distinct therapeutic consequences or a less aggressive phenotype of IDH-mutated glioma.

While multiple studies have proposed to combine PET and advanced MRI to improve diagnostic accuracy for the diagnosis of true tumour progression [38–40], each modality is usually assessed in a separate manner and quantitative endpoints are ultimately combined. Here, we showed that there is a benefit in a combined and integrated methodology where microstructure markers were determined in metabolically active tumour compartments guided by ^18^F-FET PET using 3D ROIs from automated iso-contouring — whereas most studies employ a sampling strategy based on manually drawn 2D ROIs within a contrast-enhancing lesion. In a recent retrospective study comprising 40 patients, Wu et al [41] suggested in contrast to our findings that DKI metrics are superior to DTI metrics in differentiating HGG recurrence from pseudoprogression. However, small circular ROIs were manually drawn in contrast-enhancing lesions and perilesional oedema, which could be prone to sampling error or might result in measurements that are less representative and/or objective. Dang et al [42] retrospectively evaluated ^11^C-MET PET and DKI in 86 glioblastoma patients with suspected recurrence after radiotherapy. ROIs were manually drawn in a single slice of a contrast-enhancing lesion, restricting the biological pertinence and generalisability of quantitative measurements in these heterogenous lesions. Consequently, mean rMD (AUC = 0.57) and rMK (AUC = 0.65) demonstrated limited diagnostic capability, although the kurtosis texture metrics (AUC = 0.76–0.79) facilitated slightly better differentiation between recurrence and radioinjury. Unfortunately, FA was not determined in this study. Furthermore, the study is limited by the absence of an appropriate multiple-comparison correction despite comparative analysis of a plethora of histogram features (translating into an increased risk for type 1 error and inflated statistical associations) and a short follow-up period (median 10.6 months). Nevertheless, there is a minor difference in sample size compared to the current study and metrics were evaluated in independent groups. Despite relevant differences with regard to the methodology and study population (acute vs. late post-treatment radiographic changes), the combined analysis of DKI and ^11^C-MET PET presented an improved diagnostic accuracy (AUC = 0.95), further suggesting that multiparametric analysis might provide benefit to patients with glioma.

In opposition to conventional DKI, Q space imaging [43] or diffusion spectrum imaging [44], which respectively depend on lengthy and extensive imaging protocols, the fast-DKI variant proposed by Hansen et al [17] substantially shortens acquisition time and reduces data requirements, which enables the concurrent use in clinical hybrid PET/MRI protocols. While diffusion-weighted imaging (DWI) is still widely used for follow-up imaging and DTI is usually performed for pre-operative imaging, the fast-DKI variant provides higher sensitivity towards alterations of the tissue microstructure with benefits for tumour grading [45] or the detection of relapsing cancer (this study) at the same or even shorter measurement time of clinically used diffusion protocols.

### Limitations

There were certain limitations due to retrospective data analysis. Because inconclusive CE-MRI usually results in referral for hybrid PET/MRI, a selection bias is possible. Recurrent HGG were retrospectively evaluated — therefore, data from this study may not apply to the detection of recurrent low-grade gliomas (LGG). Although the PTRE cohort comprised a small subset of cases currently classified as LGG according to the 2021 WHO classification — previously categorised as HGG according to the 2016 WHO classification — we presume a low probability of false negative classifications, given the extended follow-up period. Furthermore, no differences between PTRE with previously treated HGG and LGG were apparent. Sample size is restricted; however, neuropathological validation of imaging was mostly available (70%) and patients were examined using same PET/MRI device assuring comparability and high spatiotemporal conformance. Nonetheless, further validation in independent cohorts with prospective study design is needed.

Direct comparison to the existing literature is difficult due to methodological differences (as discussed above). However, data from this study highlights the importance of sampling strategy, suggesting a benefit of the synergistic assessment of PET and MRI.

Because the current study’s focus is the characterization of tumour microstructure using dMRI, other advanced MRI techniques, such as magnetic resonance spectroscopy (MRS) or perfusion weighted imaging (PWI), which allow insights into biochemical or perfusion alterations in CNS cancer, were not evaluated. Further studies should evaluate fractional kurtosis anisotropy [46], which was not calculated due to higher signal-to-noise (SNR) requirements [47]. MKT from the fast-DKI variant is known to be accurate and robust, but may present marginal differences to mean kurtosis (MK) computed using the traditional DKI framework [17]. Although DKI provides high sensitivity towards alterations of the tissue microstructure, diffusion metrics are not specific to the cancer pathology and need to be carefully interpreted in multimorbid patients.

## Conclusion

 Multimodal PET/MR imaging with combined and integrative analysis of ^18^F-FET PET and DKI presents clinical benefit for the assessment of CNS cancer, particularly with regard to the detection of glioma relapse. Microstructure markers of the metabolic periphery yielded more biologically pertinent estimates, characterising the tumour microenvironment, and, thereby, presented improved diagnostic accuracy for the detection of relapsing glioma with similar accuracy to amino acid PET, while multimodal biparametric analysis presented an additional benefit to the assessment based on PET alone.

### Supplementary Information

Below is the link to the electronic supplementary material.

Supplementary file1 (PDF 153 KB)

## Abbreviations

ATRX: Alpha-thalassemia/mental retardation syndrome X-linked
DKI: Diffusion kurtosis imaging
FA: Fractional anisotropy
FET: O-(2-^18^F-fluoroethyl)-l-tyrosine
HGG/LGG: High-/low-grade glioma
IDH: Isocitrate dehydrogenase
LOH1p/19q: Loss of heterozygosity on chromosomes 1p and 19q
MD: Mean diffusivity
MGMT: O6-methylguanine-DNA methyltransferase
MKT: Mean kurtosis tensor
PTRE: Post-treatment-related effects

## Declarations

### Guarantor

The scientific guarantor of this publication is Prof. Marcus R. Makowski.

### Statistics and biometry

No complex statistical methods were necessary for this paper.

### Study subjects or cohorts overlap

There is no subject overlap with previously published works.

### Methodology

• retrospective

• diagnostic or prognostic study

• performed at one institution
