# Supplementary material for: Detection of recurrent high-grade glioma using microstructure characteristics of distinct metabolic compartments in a multimodal and integrative 18F-FET PET/fast-DKI approach
Source: Eur Radiol. 2023 Sep 6;34(4):2487–99. doi: 10.1007/s00330-023-10141-0 (PMC10957712; doi:10.1007/s00330-023-10141-0)
Supplement: Supplementary file 1 — Supplementary file1 (PDF 153 KB) [file 330_2023_10141_MOESM1_ESM.pdf]

Appendix E1

Patient ID	Age (a), Sex (M/F)	WHO tumour classification	IDH mutation	MGMT promoter methylation	LOH1p/19q status	ATRX loss	Diagnosis (MRI/HIST)	Therapy-to-scan interval (mo)	Last intervention scheme
**1**	67, M	Glioblastoma IDH-WT (CNS WHO grade 4)	0	1	-	0	PTRE (MRI)	17	R, RCTx, CTx
**2**	61, M	Glioblastoma IDH-WT (CNS WHO grade 4)	0	1	-	0	PTRE (MRI)	9	R, RCTx, CTx
**3**	45, M	Astrocytoma IDH-MT (CNS WHO grade 3)	1	1	-	1	PTRE (MRI)	20	R, RCTx, CTx
**4**	50, M	Glioblastoma IDH-WT (CNS WHO grade 4)	0	1	-	0	PTRE (MRI)	6	R, RTx, CTx
**5**	49, M	Astrocytoma IDH-MT (CNS WHO grade IV)	1	1	-	1	PTRE (MRI)	30	R, RCTx
**6**	46, M	Oligodendroglioma IDH-MT, 1p/19q co-deleted (CNS WHO grade 3)	1	0	1	0	PTRE (MRI)	46	R, RCTx, CTx
**7**	30, W	Oligodendroglioma IDH-MT, 1p/19q co-deleted (CNS WHO grade 3)	1	1	1	0	PTRE (MRI)	46	R, RTx, CTx
**8**	49, W	Oligodendroglioma IDH-MT, 1p/19q co-deleted (CNS WHO grade 3)	1	1	1	0	PTRE (MRI)	62	R, CTx, RTx
**9**	23, M	Anaplastic pleomorphic Xanthoastrocytoma (CNS WHO grade 3)	0	0	0	0	PTRE (HIST)	34	R, RCTx, CTx, ICI, RTx
**10**	48, W	Oligodendroglioma IDH-MT, 1p/19q co-deleted (CNS WHO grade 3)	1	-	1	0	PTRE (HIST)	46	R, RCTx
**11**	35, W	Astrocytoma IDH-MT (CNS WHO grade 2)	1	1	0	1	PTRE (MRI)	36	R, RCTx, CTx

Appendix E1

**12**	43, W	Oligodendroglioma IDH-MT, 1p/19q co-deleted (CNS WHO grade 3)	1	1	1	0	PTRE (MRI)	38	R, CTx, RCTx
**13**	46, W	Oligodendroglioma IDH-MT, 1p/19q co-deleted (CNS WHO grade 2)	1	-	1	0	PTRE(MRI)	57	R, CTx
**14**	58, W	Oligodendroglioma IDH-MT, 1p/19q co-deleted (CNS WHO grade 2)	1	1	1	0	PTRE(MRI)	41	R, CTx
**15**	51, W	Astrocytoma IDH-MT (CNS WHO grade 2)	1	1	0	1	PTRE(MRI)	12	R, RCTx, CTx
**16**	54, M	Astrocytoma IDH-MT (CNS WHO grade 2)	1	1	-	1	PTRE(MRI)	26	R, RTx, CTx
**17**	39, M	Oligodendroglioma IDH-MT, 1p/19q co-deleted (CNS WHO grade 2)	1	1	1	0	PTRE(MRI)	24	R, CTx, RTx
**18**	47, W	Oligodendroglioma IDH-MT, 1p/19q co-deleted (CNS WHO grade 2)	1	-	1	-	PTRE(MRI)	24	R, RTx
**19**	57, W	Glioblastoma IDH-WT (CNS WHO grade 4)	0	1	-	0	PTRE(MRI)	30	R, RCTx, CTx
**20**	57, W	Astrocytoma IDH-MT (CNS WHO grade 3)	1	1	0	1	PTRE(MRI)	38	R, RCTx
**21**	57, W	Oligodendroglioma IDH-MT, 1p/19q co-deleted (CNS WHO grade 3)	1	1	1	0	REC (HIST)	87	R, CTx
**22**	49, M	Astrocytoma IDH-MT (CNS WHO grade 3)	1	1	0	1	REC (HIST)	25	R, RCTx

Appendix E1

**23**	54, W	Oligodendroglioma IDH-MT, 1p/19q co-deleted (CNS WHO grade 3)	1	-	1	0	REC (HIST)	59	RTx
**24**	50, M	Glioblastoma IDH-WT (CNS WHO grade 4)	0	0	-	0	REC (HIST)	6	R, RCTx
**25**	44, W	Oligodendroglioma IDH-MT, 1p/19q co-deleted (CNS WHO grade 3)	1	-	1	0	REC (HIST)	74	R, RCTx
**26**	61, W	Glioblastoma IDH-WT (CNS WHO grade 4)	0	0	-	0	REC (HIST)	3	R, RTx, CTx
**27**	33, W	Oligodendroglioma IDH-MT, 1p/19q co-deleted (CNS WHO grade 3)	1	-	1	0	REC (HIST)	70	R, CTx, RTx
**28**	37, W	Astrocytoma IDH-MT (CNS WHO grade 3)	1	1	0	0	REC (HIST)	39	R, RCTx
**29**	39, W	Glioblastoma IDH-WT (CNS WHO grade 4)	0	0	0	0	REC (HIST)	4	R, RCTx
**30**	34, M	Oligodendroglioma IDH-MT, 1p/19q co-deleted (CNS WHO grade 3)	1	0	1	0	REC (HIST)	65	R, RTx
**31**	64, W	Glioblastoma IDH-WT (CNS WHO grade 4)	0	1	-	0	REC (HIST)	3	R, CTx, RTx
**32**	35, M	Astrocytoma IDH-MT (CNS WHO grade 3)	1	0	0	1	REC (HIST)	96	R, RCTx
**33**	48, M	Glioblastoma IDH-WT (CNS WHO grade 4)	0	-	0	0	REC (HIST)	7	R, RCTx
**34**	33, W	Astrocytoma IDH-MT (CNS WHO grade 3)	1	-	0	0	REC (HIST)	9	R, watch-and-wait
**35**	28, M	Glioblastoma IDH-WT (CNS WHO grade 4)	0	1	0	1	REC (HIST)	84	R, watch-and-wait

Appendix E1

**36**	55, M	Oligodendroglioma IDH-MT, 1p/19q co-deleted (CNS WHO grade 3)	1	-	1	-	REC (HIST)	83	R, RCTx
**37**	59, M	Glioblastoma IDH-WT (CNS WHO grade 4)	0	1	-	-	REC (HIST)	18	R, RCTx
**38**	47, W	Astrocytoma IDH-MT (CNS WHO grade 4)	1	1	-	1	REC (HIST)	31	R, CTx, RTx
**39**	62, W	Glioblastoma IDH-WT (CNS WHO grade 4)	0	1	0	0	REC (HIST)	4	R, RCTx
**40**	62, M	Glioblastoma IDH-WT (CNS WHO grade 4)	0	1	-	0	REC (HIST)	7	R, RTx, RCTx
**41**	50, W	Glioblastoma IDH-WT (CNS WHO grade 4)	0	1	0	0	REC (HIST)	5	R, RCTx,
**42**	54, W	Anaplastic pleomorphic Xanthoastrocytoma (CNS WHO grade 3)	0	0	0	0	REC (HIST)	11	RCTx
**43**	22, W	Glioblastoma IDH-WT (CNS WHO grade 4)	0	0	0	0	REC (HIST)	6	R, RCTx
**44**	44, M	Astrocytoma IDH-MT (CNS WHO grade 3)	1	1	0	1	REC (HIST)	34	R, CTx, RTx
**45**	50, W	Astrocytoma IDH-MT (CNS WHO grade 4)	1	1	-	-	REC (HIST)	82	R, RCTx
**46**	34, M	Astrocytoma IDH-MT (CNS WHO grade 4)	1	1	0	1	REC (HIST)	6	R, RCTx
**47**	63, M	Glioblastoma IDH-WT (CNS WHO grade 4)	0	1	-	0	REC (HIST)	4	R, RCTx
**48**	43, M	Astrocytoma IDH-MT (CNS WHO grade 3)	1	1	0	1	REC (HIST)	95	R, watch-and-wait
**49**	50, M	Glioblastoma IDH-WT (CNS WHO grade 4)	0	0	-	0	REC (HIST)	7	R, CTx

Appendix E1

**50**	42, W	Astrocytoma IDH-MT (CNS WHO grade 3)	1	-	0	1	REC (HIST)	44	R, CTx
**51**	33, W	Astrocytoma IDH-MT (CNS WHO grade 3)	1	1	0	1	REC (HIST)	74	R, CTx
**52**	27, M	Astrocytoma IDH-MT (CNS WHO grade 4)	1	0	0	1	REC (HIST)	42	R, CTx
**53**	42, W	Astrocytoma IDH-MT (CNS WHO grade 4)	1	1	0	1	REC (HIST)	85	R, CTx, RTx
**54**	59, M	Oligodendroglioma IDH-MT, 1p/19q co-deleted (CNS WHO grade 3)	1	1	1	0	REC (HIST)	80	R, RTx, CTx
**55**	67, W	Glioblastoma IDH-WT (CNS WHO grade 4)	0	0	-	0	REC (HIST)	8	R, RCTx
**56**	53, M	Glioblastoma IDH-WT (CNS WHO grade 4)	0	1	-	0	REC (HIST)	20	R, RCTx
**57**	63, M	Glioblastoma IDH-WT (CNS WHO grade 4)	0	0	-	0	REC (HIST)	12	R, RCTx
**58**	72, W	Glioblastoma IDH-WT (CNS WHO grade 4)	0	0	0	0	REC (HIST)	8	R, RCTx
**59**	50, M	Glioblastoma IDH-WT (CNS WHO grade 4)	0	0	0	0	REC (HIST)	11	R, RTx, CTx

Table 1. **Demographic and clinical data.** M = male. F = female. a/mo = annum/months. WHO = World Health Organization. IDH-MT/-WT = Isocitrate dehydrogenase mutant/wildtype. MGMT = O6-methylguanine-DNA-methyltransferase. ATRX = alpha-thalassemia/mental retardation syndrome X-linked. LOH1p/19q = Loss of heterozygosity (LOH) of 1p/19q. PTRE = Post-treatment related effects. REC = Recurrent glioma. Binary classification (LOH1p/19q+, 1, LOH1p/19q-, 0; IDH-MT, 1, IDH-WT, 0; MGMT+, 1, MGMT-, 0; ATRX+, 1, ATRX-, 0). R = Resection or extended biopsy. RCTx = Concomitant radiochemotherapy. RTx = Radiotherapy. CTx = Chemotherapy. ICI = Immune checkpoint inhibitor.
